# Giant focal nodular hyperplasia with a background of hepatic steatosis in a 14-year-old boy

**DOI:** 10.1093/jscr/rjac238

**Published:** 2022-05-27

**Authors:** Andrew M Fleming, Caitlyn Duffy, Jessica Gartrell, M Beth McCarville, Max R Langham, Robert E Ruiz, Teresa Santiago, Andrew J Murphy

**Affiliations:** Department of Surgery, St Jude Children’s Research Hospital, Memphis, TN, USA; Department of Surgery, University of Tennessee Health Science Center, Memphis, TN, USA; Department of Oncology, St Jude Children’s Research Hospital, Memphis, TN, USA; Department of Oncology, St Jude Children’s Research Hospital, Memphis, TN, USA; Department of Diagnostic Imaging, St Jude Children’s Research Hospital, Memphis, TN, USA; Department of Surgery, St Jude Children’s Research Hospital, Memphis, TN, USA; Division of Pediatric Surgery, University of Tennessee Health Science Center, Memphis, TN, USA; Department of Pathology, St Jude Children’s Research Hospital, Memphis, TN, USA; Department of Pathology, St Jude Children’s Research Hospital, Memphis, TN, USA; Department of Surgery, St Jude Children’s Research Hospital, Memphis, TN, USA; Division of Pediatric Surgery, University of Tennessee Health Science Center, Memphis, TN, USA

## Abstract

Giant focal nodular hyperplasia (GFNH) is rarely seen in children, presenting complex diagnostic and management considerations. Pathognomonic radiographic findings can be absent in this population, and the nuances of pathologic examination are critical. We present a child with a GFNH involving the right side of the liver arising in the background of hepatic steatosis. The details of the diagnosis and therapeutic decisions involved in his treatment are discussed.

## INTRODUCTION

Focal nodular hyperplasia (FNH) is an uncommon diagnosis in children [1]. Lesions are typically less than 5 cm, though giant focal nodular hyperplasia (GFNH) has been documented in the literature [2–4]. Other liver masses can masquerade as GFNH [5]. On histology and MRI, FNH classically demonstrates a central stellate scar of fibrous connective tissue and can be classified into typical and atypical FNH [6, 7]. Here, we present a rare case of a GFNH in a pediatric patient.

## CASE REPORT

**Figure 1:**
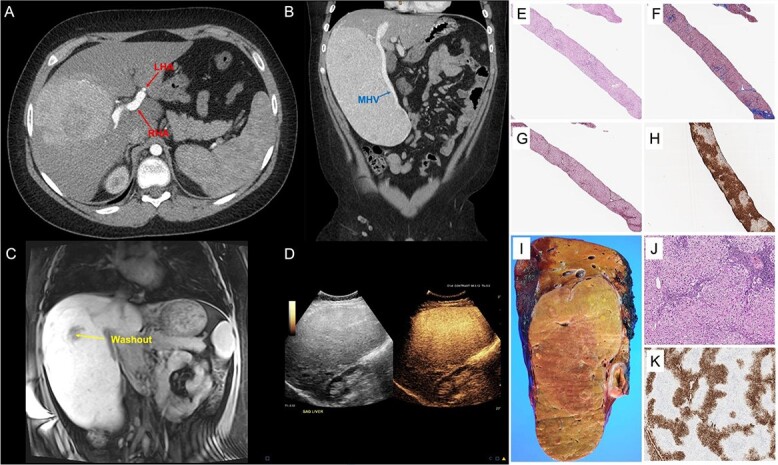
GFNH; (**A–D**) representative images from CT of the abdomen and pelvis with contrast, MRI of the liver with gadoxetate disodium and contrast-enhanced ultrasound; (**A**) the large, heterogenous mass in the right liver with an enlarged RHA and a diminutive LHA, and panel (**B**) further characterizes its exophytic nature, also demonstrating the enlarged MHV; panel (**C**) depicts a coronal section of an MRI of the liver with gadoxetate disodium showing the 14 cm × 11 cm × 21 cm, PRETEXT II lesion involving segments V, VI, VII and VIII and a small, central area of washout, and panel (**D**) shows homogenous hyperenhancement of the liver lesion on contrast-enhanced ultrasound; (**E–K**) pathological features of giant focal nodular hyperplasia; (**E–H**) needle core biopsy; proliferation of well-differentiated hepatocytes displaying a nodular architecture (**E**, Hematoxylin & Eosin, 40×) and separated by bands of fibrosis (**F**, trichrome stain, 40x and G, reticulin stain, 40×), and GS immunostain shows a geographic pattern (**H**, immunohistochemistry, 40×); (**I** and **J**) partial hepatectomy (segments 5, 6, 7 and 8) with cholecystectomy; coronal section of the right hepatectomy specimen demonstrating a tan-yellow and well-circumscribed mass measuring 22.1 × 13.0 × 6.0 cm (**I**, gross picture) with hepatocellular proliferation with nodular architecture and containing occasional abnormal vessels and ductules (**J**, Hematoxylin and Eosine, 100×); GS performed in the resection specimen also displays a geographic pattern of staining (**K**, immunohistochemistry, 40×).

A 14-year-old boy presented with abdominal pain. Abdominal ultrasonography and CT scan revealed a liver mass 21 cm in greatest dimension (Fig. 1A–D). The right hepatic artery (RHA) supplying the mass was enlarged, while the left hepatic artery (LHA) was small in caliber (Fig. 1A). The middle hepatic vein (MHV) was enlarged (Fig. 1B), and a diminutive left hepatic vein suggested vascular steal. He had elevated transaminases; alanine aminotransferase (ALT) = 129 (normal 0–40), aspartate aminotransferase (AST) = 60 (normal 0–40). Serum alpha fetoprotein (AFP) was normal; AFP = 2 (normal 0.8–12). MRI of the liver with hepatocyte-specific contrast (gadoxetate disodium) revealed a 14 × 11 × 21 cm, PRETEXT II lesion involving segments V, VI, VII and VIII [8]. Most of the mass retained contrast in the 20-minute hepatocyte phase except a small, central area that washed out (Fig. 1C). The MRI revealed underlying steatosis in the left liver and fat-containing nodules throughout the mass. Contrast-enhanced ultrasound revealed homogenous hyperenhancement (Fig. 1D) in the early arterial phase that was retained through delayed imaging. Because the etiology of the mass and the underlying liver disease remained unclear, ultrasound-guided percutaneous biopsy of the lesion and the adjacent non-neoplastic liver was performed.

Histologically, the non-neoplastic liver revealed moderate microvesicular and macrovesicular steatosis with mild inflammatory infiltrate. No significant fibrosis was noted. H&E-staining of the liver mass demonstrated proliferation of well-differentiated hepatocytes displaying nodular architecture separated by bands of fibrosis highlighted by trichrome staining. Endothelialization of sinusoids was observed with an incomplete pattern of CD34 staining. Reticulin staining emphasized that most cell plates were no more than 2–3 hepatocytes in thickness. Bile duct proliferation was noted at the periphery of the nodules, best appreciated with the aid of cytokeratin 7. Glutamine synthetase (GS) staining showed a geographic pattern with hepatocyte sparing close to the fibrous band (Fig. 1E–H). Glypican-3 and Sall4 immunostains were both negative. Beta-catenin staining showed only membranous/cytoplasmic reactivity. No aberrant nuclear accumulation of beta-catenin was observed. Based on these findings, the differential diagnosis included several well-differentiated hepatocellular lesions, including FNH, hepatic adenoma, well-differentiated hepatocellular carcinoma (HCC) and fibrolamellar HCC.

The geographic pattern of GS staining coupled with the imaging findings pointed to a probable diagnosis of FNH, although well-differentiated HCC and hepatic adenoma could not be entirely excluded. The etiology of the steatosis in this patient was unclear, but it may be associated with his increased body mass index (31.3 kg/m^2^). Given the patient’s symptoms and the possibility of malignancy, we proceeded with right hepatectomy. The patient was discharged home on post-operative Day 9 due to rehabilitation requirements, an episode of opiate-induced delirium and a surgical wound complication (seroma). Post-operative follow-up demonstrated a normalization of the patient’s AST and ALT. After unremarkable MRIs at 3 and 6 months, the patient was discharged from clinic.

Gross examination of the right hepatectomy specimen (1927 g, 24.0 × 16.5 × 6.0 cm) revealed a well-circumscribed nodular lesion (22.1 × 13.0 × 6.0 cm; Fig. 1I and J). Immunostaining for GS was performed, revealing the same map-like staining pattern observed in the biopsy (Fig. 1K). The large fibrous septae were highlighted with trichrome stain. The overall findings were consistent with FNH. Additional stains performed with negative results included liver fatty acid-binding protein (LFABP), hepatic amyloid A and c-reactive protein (CRP), ruling out the possibility of HA or malignancy.

Whole-genome, whole-exome and transcriptome analyses were performed, which revealed no reportable somatic copy number alterations, structural variants, insertion/deletion polymorphisms or single nucleotide variants, with adequate sample purity.

## DISCUSSION

Differentiating FNH from other pediatric hepatic lesions is imperative. FNH is polyclonal and is not considered a neoplastic process but rather a hyperperfusion-induced hyperplasia due to an underlying vascular anomaly. This contrasts with the monoclonal processes characteristic of hepatic adenomas [9]. An ‘imbalance’ in angiopoietin has been implicated in FNH development, contrasting with HCC [10]. Differential microRNA expression has been demonstrated in FNH, another potential diagnostic adjunct [11].

Risk factors for FNH in children are not well defined. Pillon *et al*. [12] described an association with hematopoietic stem cell transplantation. Nevertheless, the patients in that series had small, multifocal FNH. Other investigators report liver lesions in 17% of pediatric solid tumor patients, the majority of which are multifocal FNH [13]. Children comprise less than 5% of patients diagnosed with FNH [14], and optimal treatments are not defined. Zarfati *et al.* [15] suggest surgery for symptoms, significant mass effect, diagnostic uncertainty or rapid tumor growth.

FNH should be in the differential diagnosis of well-differentiated hepatocellular lesions in children, even if they are large or lack classical radiological features. Since our patient was symptomatic, and there was diagnostic uncertainty, we resected the lesion, which yielded symptomatic improvement, addressed concern for underlying malignancy and allowed for a thorough pathologic examination.

## CONFLICT OF INTEREST STATEMENT

The authors obtained IRB approval (IRB: 21–0938) and declare no conflicts of interest.
